# Supplementary material for: Causal Relationship between Aging and Anorexia Nervosa: A White-Matter-Microstructure-Mediated Mendelian Randomization Analysis
Source: Biomedicines. 2024 Aug 16;12(8):1874. doi: 10.3390/biomedicines12081874 (PMC11351342; doi:10.3390/biomedicines12081874)
Supplement: Supplementary file 1 [file biomedicines-12-01874-s001.zip › Supplementary Figure/Figure S4/Singe SNP plot of MR analysis for TL to ubm-b-2013.pdf]

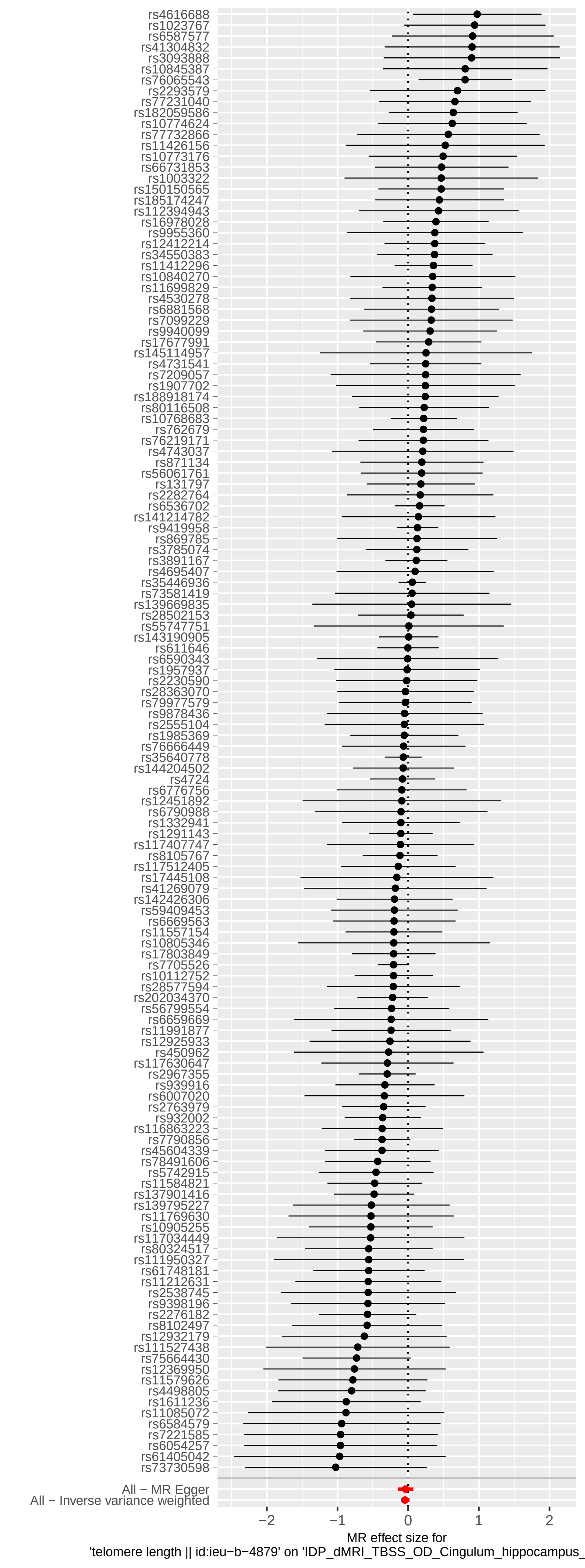
rs4616688
rs1023767
rs6587577
rs41304832
rs3093888
rs10845387
rs76065543
rs2293579
rs77231040
rs182059586
rs10774624
rs77732866
rs11426156
rs10773176
rs66731853
rs1003322
rs150150565
rs185174247
rs112394943
rs16978028
rs9955360
rs12412214
rs34550383
rs11412296
rs10840270
rs11699829
rs4530278
rs6881568
rs7099229
rs9940099
rs17677991
rs145114957
rs4731541
rs7209057
rs1907702
rs188918174
rs80116508
rs10768683
rs762679
rs76219171
rs4743037
rs871134
rs56061761
rs131797
rs2282764
rs6536702
rs141214782
rs9419958
rs869785
rs3785074
rs3891167
rs4695407
rs35446936
rs73581419
rs139669835
rs28502153
rs55747751
rs143190905
rs611646
rs6590343
rs1957937
rs2230590
rs28363070
rs79977579
rs9878436
rs2555104
rs1985369
rs76666449
rs35640778
rs144204502
rs4724
rs6776756
rs12451892
rs6790988
rs1332941
rs1291143
rs117407747
rs8105767
rs117512405
rs17445108
rs41269079
rs142426306
rs59409453
rs6669563
rs11557154
rs10805346
rs17803849
rs7705526
rs10112752
rs28577594
rs202034370
rs56799554
rs6659669
rs11991877
rs12925933
rs450962
rs117630647
rs2967355
rs939916
rs6007020
rs2763979
rs932002
rs116863223
rs7790856
rs45604339
rs78491606
rs5742915
rs11584821
rs137901416
rs139795227
rs11769630
rs10905255
rs117034449
rs80324517
rs111950327
rs61748181
rs11212631
rs2538745
rs9398196
rs2276182
rs8102497
rs12932179
rs111527438
rs75664430
rs12369950
rs11579626
rs4498805
rs1611236
rs11085072
rs6584579
rs7221585
rs6054257
rs61405042
rs73730598
All − MR Egger
All − Inverse variance weighted
−2
−1
0
1
2
MR effect size for
'telomere length || id:ieu−b−4879' on 'IDP_dMRI_TBSS_OD_Cingulum_hippocampus_
